# Rationale and design of the multicenter, national, randomized, open labeled phase III trial: allogeneic stem cell transplantation as a potential curative treatment for patients with relapsed or progressed multiple myeloma (AlloRelapseMM Study)

**DOI:** 10.1186/s12885-025-13503-7

**Published:** 2025-01-27

**Authors:** Annemarie Glöckner, Stefan Schönland, Hermann Einsele, Nicolaus Kröger

**Affiliations:** 1 Department of Stem Cell Transplantation, University Medical Center Hamburg-Eppendorf, Hamburg, Germany; 2 University Hospital Heidelberg, Medical Clinic V, Heidelberg, Germany; 3 Department of Internal Medicine II, University Hospital Würzburg, Würzburg, Germany

**Keywords:** Allogeneic stem cell transplantation, Multiple myeloma, Salvage therapy

## Abstract

**Background:**

Even though major improvements have been made in the treatment of myeloma, the majority of patients eventually relapse or progress. Patients with multiple myeloma who relapse after initial high-dose chemotherapy with autologous stem cells have a median progression free survival up to 2–3 years, depending on risk factors such as previous remission duration. In recent years, growing evidence has suggested that allogeneic stem cell transplantation could be a promising treatment option for patients with relapsed or progressed multiple myeloma. However, prospective randomized trials including allogeneic stem cell transplantation as second-line therapy do not exist to date and therefore urgently needed to demonstrate the value of this therapy in the overall setting of patients with multiple myeloma.

**Methods:**

This clinical trial is a national, multicenter, randomized, open labeled phase III study conducted in 30 hospitals spread all over Germany. After study inclusion, all patients will receive 3 cycles of salvage therapy with one of the currently approved triplet regimens for first relapse. After 3 cycles of salvage therapy, remission status will be assessed. If the patient achieves at least stable disease, partial or complete remission and an HLA compatible stem cell donor could be identified, he/she will be randomized (1:1) to the control or interventional study arm. Approximately 400 patients will be enrolled to enable a randomization of 280 patients. The primary endpoint is overall survival at five years after randomization. The main secondary objectives and endpoints are progression-free survival rate, time-to first occurrence of an infection with CTCAE grade 3–5, non-relapse mortality rate and incidence of acute and chronic graft-versus-host disease after allogeneic stem cell transplantation.

**Discussion:**

The present clinical study is designed to evaluate the superiority of allogeneic stem cell transplantation compared to conventional therapy (triplet chemotherapy) for the difference in overall survival at 5 years, toxicity and quality of life in patients with multiple myeloma who have relapsed or progressed after first-line autologous stem cell therapy.

**Trial registration:**

ClinicalTrials.gov (NCT05675319) registered on January 9, 2023.

## Background

Multiple myeloma (MM) is the second most common hematological malignancy and is considered to be an incurable disease. MM is characterized by uncontrolled plasma tumor cell proliferation, triggered by intrinsic chromosomal abnormalities and extrinsic stromal cell support. Simultaneously, monoclonal protein in the blood and/ or urine is detectable [1]. The incidence is approximately 6–8 per 100.000 people with the median age at diagnosis about 70 years. The symptoms of MM are varied and often unspecific. In some cases, symptoms persist for several months before a diagnosis is made. Today, up to 25% of patients are symptom-free at the time of diagnosis [2–4].

Initial treatment for multiple myeloma typically incorporates induction therapy followed by a high-dose chemotherapy with subsequent autologous stem cell transplantation (auto SCT) for eligible patients [5]. Even though major improvements have been made in the treatment of multiple myeloma in the last decade, the majority of patients eventually relapse or progress [6]. Patients who relapse after first-line therapy have a median progression free survival up to 2–3 years, depending on risk factors such as previous remission duration [7].

Allogeneic stem cell transplantation (allo SCT) is a potential curative treatment, but is associated with a higher risk of morbidity and treatment-related mortality compared to auto SCT due to graft-versus-host effect. On the other side, the graft-versus-myeloma effect reduces the risk of recurrence significantly compared to auto SCT or conventional therapy [8–10].

New effective drugs in the treatment of relapsed or progressed MM resulting in increasingly improved treatment outcomes. Triplet combinations and quadruplets that are approved for second-line treatment of MM in Germany, achieve a median progression-free interval of more than 35 months depending on diverse risk factors such as patient-related morbidity and tumor-related features [11]. Novel immunotherapies such as bispecific antibodies or chimeric antigen receptor (CAR) T cells have been approved more recently although in a late stage of the disease.

Prospective randomized trials including allo SCT in relapsed multiple myeloma patients do not exist to date. Retrospective donor versus no-donor comparisons showed a survival benefit for allo SCT, but a strong selection bias cannot be ruled out [12]. Randomized trials with allo SCT beyond first-line therapy are therefore urgently needed to demonstrate the value of this therapy in the overall setting of patients with multiple myeloma.

Within this AlloRelapseMM study the superiority of allo SCT versus conventional therapy as salvage therapy in patients with relapsed or progressive multiple myeloma after first-line therapy shall be demonstrated. After study inclusion, all patients receive an salvage therapy consisting of 3 cycles (treatment phase 1). Patients who have not became progressive in treatment phase 1 will be randomized either to receive an allo SCT or to continue the salvage therapy. This clinical trial will be the first prospective randomized phase III trial investigating the importance and benefit of allo SCT regarding overall survival (OS), progression-free survival (PFS) and quality of life in patients diagnosed with multiple myeloma.

## Methods

### Study design

In this clinical trial, allo SCT in patients diagnosed with multiple myeloma beyond first-line therapy will be tested. It is a national, multicenter, randomized, open labeled phase III study. Blinding is not possible due to different patient treatments in both study arms. In total 280 patients will be randomized at a 1:1 ratio either to receive the allo SCT (Arm A) or the conventional therapy (Arm B). To achieve this sample size, approximate 482 patients have to be screened.

### Trial objectives

#### Primary objective

The present clinical study aims to demonstrate the superiority of allo SCT compared to conventional therapy for the difference in overall survival at 5 years in patients with multiple myeloma who have relapsed or progressed after first-line auto SCT. Patients will be observed from randomization until database lock for final analysis and OS rate calculated at 5 years after randomization.

#### Secondary objectives

The secondary objectives of this trial are to compare both treatment arms (allo SCT versus conventional therapy) at 1 year, 3 years and 5 years after randomization concerning:

o Event free survival (EFS) – defined as time from randomization to first occurrence of progression, relapse, engraftment failure or death of any cause
o Changes in quality of life using patient self-filled questionnaires from the European Organization of Research and Treatment of Cancer Quality of Life Questionnaires (EORTC- QLQC30) including the Multiple Myeloma Module (EORTC- QLQMY20)
o Non-relapse mortality (NRM) – defined as time from randomization to patient’s death before any relapse reported
o Toxicity - defined as time to first occurrence of infection with CTCAE grade 3-5

In addition, the time to partial or complete remission after randomization will be assessed. For patients who will receive the allo SCT, the cumulative incidence of acute and chronic graft-versus-host disease (GvHD) will be reported.

### Study setting

This study will be conducted on behalf of and funded by the German institution Federal Joint Committee (Gemeinsamer Bundesausschuss, G-BA) to obtain significant results for the benefit assessment of allo SCT in MM beyond first-line therapy. 30 hospitals spread all over Germany, which are highly experienced in allo SCT and conduction of clinical trials, participate in this study.

### Estimated timeline

The overall duration of this study is expected to be approximately 10 years starting from site initiation visit whereby 4.5 years are calculated for the recruiting phase. The duration of intervention for each patient will be 67 months in maximum including a 60 months follow-up phase. The preparation phase of this study took 3 years. Recruitment of patients has been started in March 2023.

Planned study timelines:

o Total study duration: ca. 120 months
o Recruitment period: approx. 54 months
o Duration of intervention per patient: max. 67 months
o First Patient First Visit (FPFV): March 2023
o Last Patient First Visit (LPFV): September 2027
o Last Patient Last Visit (LPLV): April 2033
o Data Base Lock (DBL): July 2033
o Clinical Study Report (CSR) completed: December 2033

### Ethical aspects and patient protection

The study is conducted in conformity with the declaration of Helsinki (latest version) and applicable national laws and regulations. The protocol is written and the study will be conducted according to the ICH Harmonized Tripartite Guideline for Good Clinical Practice, issued by the European Union. Among other documents, the study protocol, the patient information sheet and the informed consent form were provided to all involved ethics committees and requested for approval. The leading ethics committee and local ethics committees of all participating study sites gave their approval prior to the start of the trial.

Patients have to give their written informed consent before any study related intervention will be performed. The informed consent form have to be personally signed and dated by the patient, or a legally acceptable representative, and by the physician who conducted the informed consent discussion. Patients are free to withdraw from the study at any time without giving any reason.

According to ICH E6 (Good Clinical Practice) an independent Data Safety Monitoring Board (DSMB) was established before first patient was enrolled. The DSMB is responsible for assuring the safety and interests of the trial participants and assessing the safety and efficacy of the interventions during the trial.

In addition, a continuous benefit-risk assessment based on all available safety data and any issues that may change the benefit-risk ratio is implemented.

All reported adverse events (AEs) are recorded in the electronical database system (Case Report Form). The responsible investigator will perform an assessment regarding relatedness and expectedness to the study medication, severity and intensity of an AE based on patient’s symptoms and according to the current active version of National Cancer Institute Common Terminology Criteria for Adverse Events (CTCAE). All of the adverse events, which are by definition serious adverse events (SAEs), have to be reported to the study administration without any delay, but not later than 24 h after awareness. If a suspected unexpected serious adverse reaction (SUSAR) occurs it will be reported within 7 calendar days after detection in case of a fatal or life-threatening SUSAR whereas a non-fatal and non-life-threatening SUSAR will be reported to the institutional review boards, the ethics committees and to all investigators within maximum 15 calendar days.

### Selection of study population

In principle, a clear definition of inclusion and exclusion criteria in the context of prospective randomized trials is necessary to avoid potential bias or unequal distribution in the study arms.

Patients with confirmed diagnosis of relapsed or progressed multiple myeloma after first-line therapy based on the criteria established by the International Myeloma Working Group (IMWG) are allowed to participate in this study. Additionally, the need for cancer treatment have to be fulfilled according to the SLiM-CRAB-criteria.

An age restriction of 18 to 65 years was implemented. Patients beyond the age of 65 years up to 70 years can be included in this study if the comorbidity index according to Sorror is 0 and ECOG ≤ 1. This is based on a significantly higher morbidity, but also mortality, especially after allo SCT.

Furthermore, administrating of one cycle of salvage therapy prior to study inclusion is allowed whereby it does not matter which regime is used.

Before patient`s randomization, a fully compatible stem cell donor defined as HLA-identical sibling or 10/10 matched unrelated donor (MUD) or 9/10 mismatched unrelated donor (MMUD), if mismatch affects DQB, have to be available as well as a good response after 3 cycles of salvage therapy of at least stable disease (SD), partial response (PR) or complete response (CR) have to be achieved.

The exclusion criteria were based on the expected increased morbidity and mortality. Therefore an insufficient organ function of the liver measured by a normal value for transaminases and bilirubin above three times or a severe cardiac dysfunction with a cardiac ejection fraction less than 50% lead to study exclusion. Furthermore, patients with an insufficient renal function defined as a creatinine clearance less than 30 ml/ min as well as a pulmonary function restriction (DLCO < 35%) or continuous oxygen dependency are not allowed to participate in this study. Active hepatitis B and C infections or other active malignant diseases have to be excluded before study inclusion.

If the patient has a progressive disease (PD) after 3 cycles of salvage therapy or no suitable donor could be identified before randomization, he/ she will be withdrawn from the study.

All study-specific inclusion and exclusion criteria are listed in Table 1.

**Table 1:** Inclusion and exclusion criteria of the AlloRelapseMM trial

Inclusion criteria	Exclusion criteria
Patients eligible for study inclusion/enrollment must meet criteria 1–7 and all of the criteria (1–9) before randomization: 1. Multiple Myeloma 2. Age 18—65 years 3. A signed informed consent form must be obtained before participation in the study 4. Age 66—70 years, if comorbidity index according to Sorror score = 0 and ECOG ≤ 1 5. 1st relapse/ progression according to IMWG criteria after first-line therapy (consisting of induction therapy followed by autologous transplantation once or twice and maintenance therapy) Additionally: meeting the need for treatment based on the SLiM-CRAB-criteria 6. Negative pregnancy test in female patients 7. Maximum of 1 cycle salvage therapy prior to study inclusion/enrollment 8. Availability of a fully compatible stem cell donor (HLA-ident. sibling or 10/10 MUD or 9/10 MMUD if mismatch affects DQB) after 3 cycles salvage therapy 9. CR/ PR or SD according to IMWG criteria after 3 cycles salvage therapy within the study	Patients are not included in the study if any one of criteria 1–6 are met and if criterion 7 is met before randomization: 1. Non-sufficient organ function defined as: • Bilirubin (in the absence of Meulengracht's disease), GPT or GOT ≥ 3 times higher than normal values • Cardiac ejection fraction ≤ 50% • GFR < 30 ml/ min • DLCO < 35% or continuous oxygen dependency 2. Active hepatitis B or C infection or uncontrolled HIV infection 3. Other, active malignant disease 4. Prior treatment with allogeneic stem cells 5. Participation in a clinical trial or taking an IMP within 30 days or five times the half-life of the IMP, whichever is longer, prior to study inclusion/enrollment 6. Positive serum pregnancy test at screening and before first treatment or breastfeeding 7. Progressive disease (PD) on salvage therapy

### Trial procedures

#### Screening phase

After participant's written informed consent (study registration), all patients have to undergo a screening procedure including physical examination, demographics, medical and medication history, previous cancer treatments as well as assessment of the ECOG-performance status and co-morbiditiy index. Before a patient will be enrolled in this trial, following laboratory investigations are necessary to ensure patient`s eligibility: transthoracic cardiac echocardiogram (Echo) and pulmonary function test (Lufu), biochemistry including analysis of creatinine, electrolytes, GOT, GPT, LDH, alkaline phosphatase, gamma GT, total bilirubin, uric acid, quantitative immunoglobulin, electrophoresis, immunofixation of total protein and if required pregnancy test. The measurement of free light chains in serum and 24-h collected urine will be performed to confirm or exclude monoclonality and to identify the heavy chain type and the light chain class. A bone marrow puncture has to be performed for bone marrow histology and minimal residual disease (MRD) analysis by flow cytometry. More details are listed in the visit schedule (Appendix 1, Table 3).

Finally, eligible patients should be enrolled immediately and the donor search should be initiated (Appendix 1, Table 3, study inclusion/enrollment).

#### Treatment phase 1/ induction therapy

**Fig. 1:**
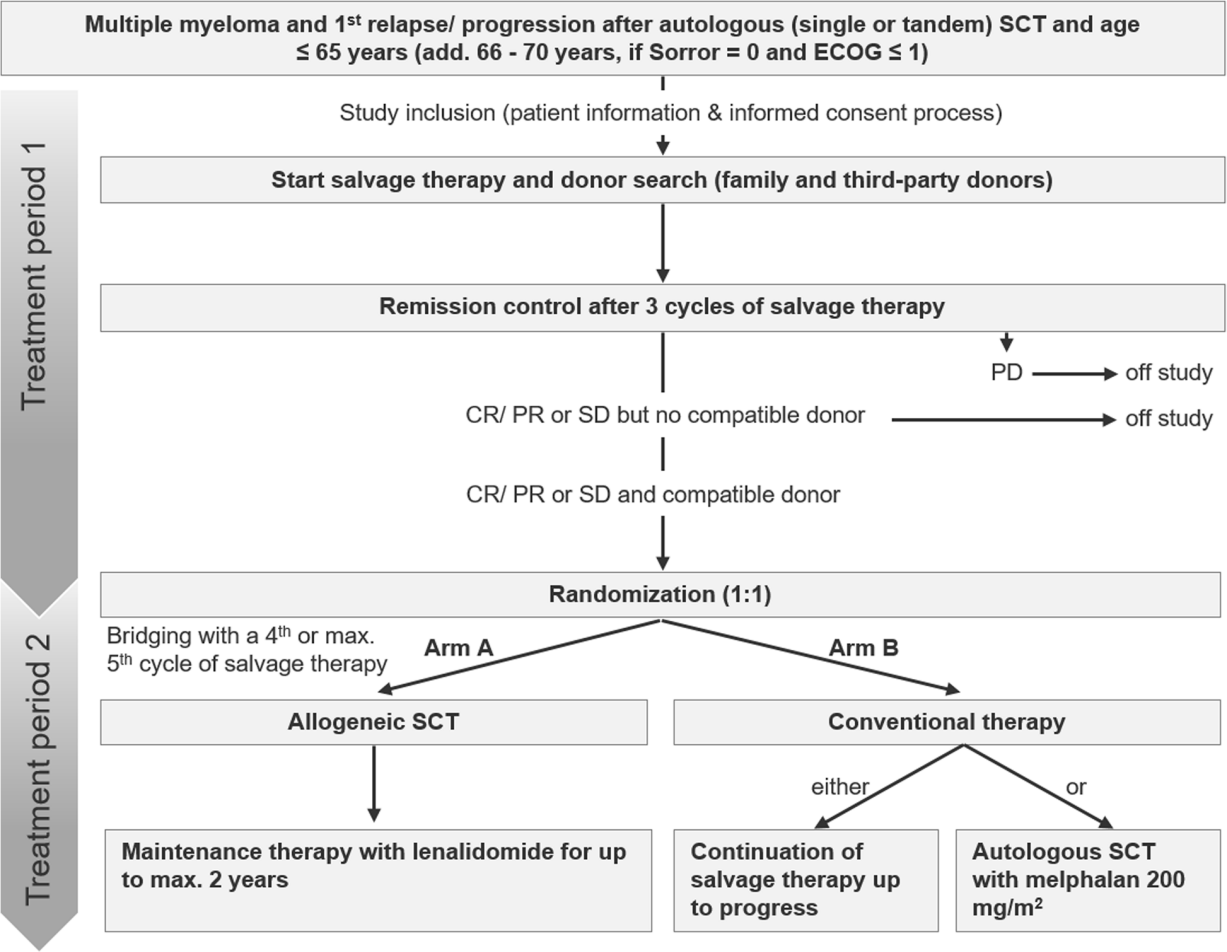
Overview of study design

After successful study enrollment, all patients will receive 3 cycles of salvage therapy as induction therapy with one of the currently approved triplet regimens for the treatment of relapsed/ progressed multiple myeloma in Germany (Fig. 1). The choice of salvage treatment combination is patient-specific and depends on patient-tumor and treatment-related features.

The following approved triplet therapies for first relapse in MM are allowed to be administrated in this trial:

o Carfilzomib/ lenalidomide/ dexamethasone (KRD) or
o Daratumumab/ lenalidomide/ dexamethasone (DRD) or
o Daratumumab/ bortezomib/ dexamethasone (DVD) or
o Daratumumab/ pomalidomide/ dexamethasone (DPD) or
o Ixazomib/lenalidomide/ dexamethasone (IRD) or
o Elotuzumab/ lenalidomide/ dexamethasone (ERD) or
o Pomalidomide/ bortezomib/dexamethasone (PVD) or
o Carfilzomib/ daratumumab/ dexamethasone (KDD) or
o Isatuximab/ carfilzomib/ dexamethasone (Isa-KD) or
o Selinexor/ bortezomib/ dexamethasone (SVD)

All treatment combinations will be administered according to latest version of the summary of product characteristics (SmPC) with respect of scheduling and dosing.

Once started, a change of salvage regimen for a single patient is not permitted.

After initiation of salvage therapy, an HLA compatible stem cell donor is sought first in the family, then an matched unrelated donor will be searched in international donor registries, if applicable.

After 3 cycles of salvage therapy, response status is assessed according to the IMWG criteria (Appendix 2, Table 6). If the patient has a PD or no suitable donor is available, he/ she will be withdrawn from the study. If the patient has achieved SD, PR or CR and a compatible stem cell donor could be identified, he/she will be randomized to the allogeneic (Arm A) or conventional (Arm B) study arm (Fig. 1).

#### Randomization and stratification

The study is conducted as a randomized study with randomization ratio 1:1 between the interventional (Arm A: allo SCT) and control arm (Arm B: conventional therapy). Blinding is not possible due to the differences between the two treatment arms. Therefore, the study is conducted as a randomized open-label study.

After 3 cycles of salvage therapy in treatment phase 1, patients will be randomized if a suitable HLA compatible donor was found and the response to induction therapy is at least SD or better. Randomization will be performed in blocks and will be stratified. Stratification will be based on the following criteria:

o Duration of remission (≤ 18 month vs. > 18 months after first auto SCT)
o Depth of remission (≥ VGPR vs. PR/ SD) after 3 cycles of salvage therapy
o Age (< or ≥ 55 years)

The study center is not used as stratification factor as the expected number of patients per center differs widely and this would therefore lead to too many incomplete blocks. Since all study centers are special clinics—carefully selected—no study center effect is expected.

The randomization list will be generated using a validated system, which involves a pseudo-random number generator so that the resulting treatment assignments will be both reproducible and non-predictable. The block size will be documented in the Clinical Study Report. Access to the codes will be controlled by access rights and trailed. Randomization will be performed according to the statistician facility’s Standard Operating Procedure.

#### Treatment phase 2/ Investigational medicinal product treatment

Patients randomized in Arm A will be treated with the test investigational medicinal product (IMP)—human allogeneic stem cells from peripheral blood. A 4^th^ and max. 5^th^ cycle of salvage therapy can be performed as bridging until preparation of stem cell transplantation is done (Fig. 1). In this case, remission status after the 4th or 5th cycle has to be confirmed (Appendix 1, Table 4).

Table 2 shows all conditioning regimes listed in the recent protocol version 5.0 that can be used within this study. All participating sites must commit to one dose-intensive and one dose-reduced treatment protocol prior to the start of the clinical trial. This procedure is intended to minimize the number of variables that could have an impact on the statistical analysis.

**Table 2:** Dose-intensive and dose-reduced condition protocols

**Dose-intensive conditioning protocols**								
						**Day**		
**Medication**	**Dose**	**-6**	**-5**	**-4**	**-3**	**-2**	**-1**	**0**
Thiotepa	5 mg/kg	x	x					
Busulfan	3.2 mg/kg			x	x	x		
								SCT
						**Day**		
**Medication**	**Dose**	**-6**	**-5**	**-4**	**-3**	**-2**	**-1**	**0**
Busulfan	3.2 mg/kg	x	x	x				
Cyclophosphamide	60 mg/kg					x	x	
								SCT
						**Day**		
**Medication**	**Dose**	**-6**	**-5**	**-4**	**-3**	**-2**	**-1**	**0**
TBI	2 Gy		2 x	2 x				
Cyclophosphamide	40 mg/kg				x	x		
								SCT
**Dose-reduced conditioning protocols**								
						**Day**		
**Medication**	**Dose**		**-5**	**-4**	**-3**	**-2**	**-1**	**0**
Thiotepa	5 mg/kg		x	x				
Busulfan	3.2 mg/kg				x	x		
								SCT
						**Day**		
**Medication**	**Dose**	**-6**	**-5**	**-4**	**-3**	**-2**	**-1**	**0**
Treosulfan	12 g/m^2^	x	x	x				
Fludarabine	30 mg/m^2^	x	x	x	x	x		
								SCT
						**Day**		
**Medication**	**Dose**	**-6**	**-5**	**-4**	**-3**	**-2**	**-1**	**0**
Melphalan	140 mg/m^2^				x			
Fludarabine	30 mg/ m^2^	x	x	x	x	x		
								SCT

According to protocol, a fixed minimum dose of 4 × 10^6^ CD34 + cells per kg recipient's body weight is mandatory. GvHD prophylaxis is given with cyclosporine A and mycophenolate mofetil/ short-term methotrexate (MTX according to institutional standard) and in case of unrelated donors additionally with ATLG. Post-transplant cyclophosphamide instead of ATLG as GvHD prophylaxis is also allowed. Subsequently to allo SCT, maintenance therapy is provided after cessation of immunosuppression with lenalidomide for a maximum of 2 years (Fig. 1).

After transplantation, the engraftment defined as an absolute neutrophil count > 0.5 × 10^9^/ l on the first day of three consecutive days will be measured as well as the chimerism analysis will be determined locally on mononuclear cells (peripheral blood mononuclear cells) from peripheral blood at every visit following SCT (Appendix 1, Table 4). Furthermore, acute and chronic GvHD will be evaluated and treated according to the standard practice procedures or the institutional guidelines of the participating institutions.

Supportive treatments such as blood cell replacement and anti-infective prophylaxis are permitted and will be performed according to institutional standards. Each site has to decide on one strategy for all their patients included. Required supportive medications for optimal medical care will be given throughout the study in accordance with institutional standards.

Human autologous stem cells from peripheral blood may be used as a comparator IMP in Arm B in this clinical trial, if sufficient stem cells are still cryopreserved. A fixed minimum dose of 2 × 10^6^ CD34 + cells per kg recipient's body weight is mandatory. Conditioning will be performed with melphalan 200 mg/ m^2^ (Fig. 1).

For the majority of patients randomized in Arm B the salvage therapy from treatment phase 1 will be continued until progression or the primary endpoint is reached, whatever comes first. Once started, a change of salvage regimen for a single patient is not permitted. Only currently approved triple regimens for the treatment of first relapsed MM can be administrated (see above, treatment phase 1). Trial visits should be scheduled at the end of a salvage therapy cycle or before the next cycle begins within a given time frame (Appendix 1, Table 5).

#### Response assessment

Response evaluation to a given treatment is one of the most important assessment in this clinical trial to compare the efficacy of Arm A (allo SCT) and Arm B (conventional therapy). Peripheral blood, urine and bone marrow will be analyzed to evaluate the response via measurement of free light chains, performing immunofixation in serum and urine including 24-h collected urine as well as conducting a minimal residual disease (MRD) analysis from bone marrow samples by flow cytometry. Evaluations will be performed in accordance to the International Myeloma Working Group (IMWG) Uniform Response Criteria for Multiple Myeloma (Appendix 2, Table 6) and the study schedule (Appendix 1, Tables 3, 4 and 5). The response to a treatment period will be assessed before the start of the subsequent period.

Patients who respond with a PD before randomization will be excluded and further treated outside of the study (Fig. 1). However, all patients that have been previously randomized in the study and discontinue from the study prematurely because of progression, relapse or graft failure will be followed at least until reaching the primary endpoint (5 years).

### Study termination

The trial may terminate at any time if serious safety concerns rise for the patients or a change in the benefit-risk ratio indicates a general increased risk for study’s participations.

#### Drop-out of single patients

Patients can withdrawal their participation at any time, even without giving a reason, without incurring any disadvantages in their medical treatment.

The local investigator can terminate the patient's participation in the study prematurely at any time for medical, safety or ethical reasons, e.g. if further treatment could be harmful or disadvantageous to the patient or if there is evidence of disease progression. Furthermore, significant protocol deviations or patient’s incompliance may also lead to a premature discontinuation.

However, it should be considered that even in case of withdrawal of treatment it is possible to continue the observation of the patient and thus to keep her or him in the study for the primary analysis.

#### Criteria for termination of single sites

An early closure of single sites is conceivable if the trial site maintained an inactive status or failed the recruiting goals immensely. In addition, individual trial sites can be closed if the data quality is not sufficient and a re-training and/ or support by the local monitor does not lead to any improvement.

#### Criteria for termination of the entire study

The sponsor and/ or coordinating principal investigator and/ or the DSMB may terminate the entire study in the event of the occurrence of excessive toxicities.

The study will be terminated early if one of the following criteria are met:

o More than 40% of study participants have organ toxicity according to NCI CTCAE guidelines (CTC grade 3 and 4), excluding transiently elevated levels of transaminases, gamma-GT, AP, and bilirubin, or
o More than 50% of patients die without disease relapse, or
o More than 20% of patients randomized into arm A experience graft failure

If the risk–benefit ratio changes to a less favorable one during the course of the study, the study may be suspended or terminated by the sponsor. In addition, the sponsor reserves the right to terminate the study for the following reasons:

o Significant failure to meet the planned recruiting goal, or
o Critical violation of GCP requirements, the study protocol, or the contract by the study site/ investigator, or
o A general disruption of the proper conduct of the study

Based on results of the planned interim analysis, which is performed after termination of recruiting phase and if approximately 75% of all included patients were observed for at least 3 years within this study, a re-evaluation of the study will be made.

### Statistical analysis

#### Sample size calculation and power considerations

Assuming a difference of 10% in overall survival at 5 years after randomization, 266 evaluable patients are needed for a power of 80% and a two-sided significance level of 5% based on a z-test on Kaplan–Meier rates (assuming a sigma of 0.29). Taking drop-outs after randomization into account (about 5%) results in 280 patients for both treatment arms (about 140 per arm). Due to the study design, it is expected that about 30% of the patients will not meet the randomization criteria at the time of randomization. Therefore, 280/0.7 = 400 patients need to be included in the study and about 482 patients need to be screened.

In the sample size considerations, drop-outs were accounted in three different ways. Firstly, patients drop-out if they do not meet the randomization criteria, which leads to the biggest drop-out of patients. Secondly, early study terminations are also considered as drop-outs, only few drop-outs (5%) are expected after randomization, since the number of study terminations generally decreases over time in an ongoing study. Thirdly, the standard deviation of the Kaplan–Meier rates also allows for drop-outs. Therefore, a total of 266 evaluable patients out of 400 included patients are considered realistic.

#### Interim analysis

An interim analysis will be performed once the last subject has been recruited and, in addition, 75% of the study participants have been followed up for 3 years after randomization or are progressed or are deceased or are lost to follow-up, whatever comes first. As the interim analysis will be exploratory in nature, the significance level on study level is not adjusted and no stopping criterion for efficacy is specified for the interim analysis. The focus of the interim analysis is the exploratory evaluation of EFS at 3 years after randomization (key secondary endpoint).

Nevertheless, a re-evaluation concerning study design, efficacy and toxicity will be made after interim analysis. The results will be discussed with the G-BA and DSMB to decide how to proceed with this clinical trial.

#### Statistical methods

All details of the biometric analyses will be specified in the statistical analysis plan (SAP), which will be completed before closure of the database and has to be authorized by the responsible statistician(s) and the responsible project manager. Any deviation from the original statistical analysis plan will be in addition to the “per protocol” analysis and will be reported as such in the protocol and/ or in the final report, as appropriate.

Descriptive statistics, including arithmetic mean, standard deviation, minimum, median and maximum, or absolute and relative frequencies will be provided according to the type of variable. For comparisons between groups, appropriate tests will be carried out at a two-sided significance level of 0.05 and confidence intervals will be presented.

Analyses will be performed with SAS® (version 9.4 or later) and/ or R (version 4.0.3 or later).

#### Analysis of primary endpoint

The treatment efficacy will be assessed in terms of the primary endpoint specified as “overall survival at 5 years after randomization”.

The superiority of the test IMP (allogeneic stem cells) will be evaluated as follows. Firstly, Kaplan–Meier estimates for survival functions will be calculated for both treatment arms and for each stratum to determine the survival rates at five years after randomization. Secondly, the standard errors for these estimates will be calculated with Greenwood’s formula.

Finally, a stratified Z-test for the absolute value of the difference of the estimates will be conducted with a two-sided significance level of α = 5%. The difference in the Kaplan–Meier estimates at 5 years after randomization will be calculated as the weighted average of the estimated Kaplan–Meier probability differences within each stratum. The weights will be defined as the "Mantel–Haenszel" weights, using the derivation as described in Greenland and Robins [13]. The variance for the overall risk difference will be calculated accordingly.

The following pair of hypotheses will be tested with S_1_ and S_2_ denoting the survival rate at 5 years in the allo SCT arm and in the control arm, respectively. Moreover, the study result will only be considered positive if a difference in survival rates in favor of the allo SCT arm is discovered (i.e., S_1_ > S_2_).

H_0_: There is no difference in overall survival at 5 years after randomization between the allo SCT and the control arm (S_1_ = S_2_).

H_1_: There is a difference in overall survival at 5 years after randomization between the allo SCT and the control (S_1_ ≠ S_2_).

To compare both treatment arms in terms of the primary endpoint a rate test is preferred to be used as the survival curves are expected to cross over between 2 and 3 years after randomization due to an increased treatment-related mortality in the allo SCT arm [14]. A relevantly higher survival rate of the test arm is expected to be detectable at 5 years after randomization, at the earliest.

The primary efficacy analysis will be performed on the Intention-to-treat (ITT) population containing all included patients who were randomized to one of the two treatment arms.

## Discussion

MM is the second most common hematological malignancy and is considered to be an incurable disease. Even though immense improvements in the treatment of MM have been made in the last years, the majority of patients relapse or progress [6].

Allo SCT is a potential curative treatment for patients diagnosed with MM. The likelihood of relapse after allo SCT is lower compared to conventional triple regime therapy or auto SCT due to the graft-versus-myeloma effect, but at the same time associated with significantly higher morbidity and transplant-related mortality [15–17].

Most patient data on allo SCT as salvage therapy after first-line therapy come from individual clinics or from registries. Due to the heterogeneity of patients, different conditioning therapies and GvHD prophylaxis, the significance and the curative potential of allo SCT in this setting cannot be conclusively assessed. Randomized trials of allo SCT beyond first-line therapy are therefore urgently needed to demonstrate the value of this therapy regarding PFS as well as OS of relapsed or progressed patients.

This study aims to demonstrate the superiority of allo SCT versus conventional therapy (approved triple therapy or auto SCT) as salvage therapy in patients with relapsed/ progressive MM after first-line therapy on the basis of 10% increase in overall survival at 5 years.

## Data Availability

No datasets were generated or analysed during the current study.

## Appendix 1

**Table 3:** Schedule of Screening and treatment phase 1

**Visit Name**	**Registration** **(patient information and informed consent form)**	**Screening Phase** ^**a**^	**Study Inclusion/Enrollment**	**End C1**	**End C2**	**End C3**
**Visit window**		Within 10 working days after registration	Within 11 working days after registration at the latest	+/- 5 d	+/- 5 d	- 10 d
**Informed Consent**	**X**					
**Inclusion/ Exclusion Criteria**		**X**				**X**
**Randomization**						**X**
**Medical history** (incl. 1^st^ line therapy consisting of induction therapy, single or double autologous transplantation and maintenance therapy)		**X**				
**Demographics**		**X**				
**P****h****ysical examination**		**X**		**X**	**X**	**X**
**Vital signs, weight, height** ^b^		**X**		**X**	**X**	**X**
**ECOG ** p**erformance status**		**X**				**X**
**Co-morbidity index**		**X**				**X**
**Cardiac evaluation/ Echo**		**X**				**X**
**Pulmonary function/ Lufu**		**X**				**X**
**B****l****ood count/ WBC diff.**		**X**		**X**	**X**	**X**
**Questionnaires **(EORTC QLQ-C30 & QLQ-MY20)		**X**				**X**
**B****o****n****e marrow investigation **(including MRD analysis at UKSH central lab Kiel)		**X**				**X**
**Serum pregnancy test** ^c^		**X**				**X**
**Blood b****i****oc****h****e****m****istry** ^d^		**X**		**X**	**X**	**X**
**Virology** ^e^		**X**				**X**
**Serum free light chain assay and serum electrophoresis**		**X**		**X**	**X**	**X**
**Serum and urine immunofixation**		**X**		**X**	**X**	**X**
**Free light chains (24 h urine)** ^f^		**X**		**X**	**X**	**X**
**HLA typing/ Donor Search**			**X**			
**Response evaluation**^g^				**X**	**X**	**X**
**Adverse events (AEs)/ Serious Adverse Events (SAEs)** ^h^		**X**		**X**	**X**	**X**
**Study and Concomitant Medication**				**X**	**X**	**X**

^a^Results of standard examinations (such as Echo, Lufu, bone marrow examination) that were routinely performed prior to registration can be used, but must not be older than 28 days prior to registration

^b^Assessment of height only at Screening

^c^For all female patients of childbearing age

^d^Creatinine, electrolytes, GOT, GPT, LDH, alkaline phosphatase, gamma GT, total bilirubin, uric acid, quantitative immunoglobulin, electrophoresis and total protein, GFR

^e^HIV, CMV, EBV, HTLV 1 and hepatitis A, B, C and E

^f^Light chains in 24 h urine at preliminary examination. Subsequently, exclusively for light chain myeloma (type Kappa or Lambda)

^g^Peripheral blood and bone marrow (bone marrow after the end of cycle 3)

^h^No AE and SAE reporting to the safety department of the CRO necessary

**Table 4:** Schedule of treatment phase 2, Arm A and Arm B (only for auto SCT)

**Visit Name**	**End C4/ C5** (optional cycles for SCT preparation)	**arm A **(allo SCT**) ** **& ** **arm B **(only if auto SCT)	**+30 d **	**+100 d**	**+ 6 mo**	**+12 mo**	**+18 mo**	**+24 mo**	**+30 mo**	**+ 36 mo**	**+48 mo**	**+60 mo**
			after SCT									
**Visit window**	- 10 d	before conditioning	+/- 5 d	+/- 7 d	+/- 30 d	+/- 30 d	+/- 30 d	+/- 30 d	+/- 30 d	+/- 30 d	+/- 30 d	+/- 30 d
**P****h****ysical examination**		**X**	**X**	**X**	**X**	**X**	**X**	**X**	**X**	**X**	**X**	**X**
**Vital signs, weight**		**X**	**X**	**X**	**X**	**X**	**X**	**X**	**X**	**X**	**X**	**X**
**Cardiac evaluation/ Echo**						**X**		**X**		**X**	**X**	**X**
**Pulmonary function/ Lufu**						**X**		**X**		**X**	**X**	**X**
**B****l****ood count/ WBC diff.**		**X**	**X**	**X**	**X**	**X**	**X**	**X**	**X**	**X**	**X**	**X**
**Questionnaires **(EORTC QLQ-C30 & QLQ-MY20)				**X**	**X**	**X**		**X**		**X**	**X**	**X**
**B****o****n****e marrow investigation **(including MRD analysis at the UKSH central lab Kiel)				**X**		**X**		**X**		**X**	**X**	**X**
**Blood b** **i** **oc** **h** **e** **m** **istry** ^a^	**X**	**X**	**X**	**X**	**X**	**X**	**X**	**X**	**X**	**X**	**X**	**X**
**Virology** ^d^	**X**											
**Serum free light chain assay and serum electrophoresis**	**X**	**X**	**X**	**X**	**X**	**X**	**X**	**X**	**X**	**X**	**X**	**X**
** Serum and urine immunofixation**		**X**	**X**	**X**	**X**	**X**	**X**	**X**	**X**	**X**	**X**	**X**
**Free light chains (24h urine)** ^**b**^		**X**	**X**	**X**	**X**	**X**	**X**	**X**	**X**	**X**	**X**	**X**
**Chimerism analysis **(only for allo SCT)			**X**	**X**	**X**	**X**	**X**	**X**	**X**	**X**	**X**	**X**
**Response evaluation** ^c^			**X**	**X**	**X**	**X**	**X**	**X**	**X**	**X**	**X**	**X**
**Adverse events (AEs)/ Serious Adverse Events (SAEs)**	**X**	**X**	**X**	**X**	**X**	**X**	**X**	**X**	**X** ^**e**^	**X** ^**e**^	**X** ^**e**^	**X** ^**e**^
**Study and Concomitant Medication**		**X**	**X**	**X**	**X**	**X**	**X**	**X**	**X**	**X**	**X**	**X**
**Engraftment **			**X**									
**Acute and chronic GvHD Evaluation **(only for allo SCT)			**X**	**X**	**X**	**X**	**X**	**X**	**X**	**X**	**X**	**X**
**remission status confirmation **(after 4^th^ or 5^th^ cycle)	**X**	**X**										

^a^Creatinine, electrolytes, GOT, GPT, LDH, alkaline phosphatase, gamma GT, total bilirubin, uric acid, quantitative immunoglobulin, electrophoresis and total protein, GFR

^b^Exclusively in light chain myeloma (kappa or lambda type)

^c^Peripheral blood and bone marrow (bone marrow only at 100 days, 12 months, 24 months, 36 months, 48 months and 60 months)

^d^HIV, CMV, EBV, HTLV 1 and hepatitis A, B, C and E

^e^SAE reporting to the Safety Department of the CRO only takes place if the SAE is presumably related to the investigational medicinal product

**Table 5:** Schedule of treatment phase 2, Arm B (continuation of salvage therapy)

**Arm B **(continuation of salvage therapy)										
**Visit Name**	**+30 d **	**+100 d**	**+ 6 mo**	**+12 mo**	**+18 mo**	**+24 mo**	**+30 mo**	**+ 36 mo**	**+48 mo**	**+60 mo**
	after start of C4									
**Visit window**	+/- 5 d	+/- 7 d	+/- 30 d	+/- 30 d	+/- 30 d	+/- 30 d	+/- 30 d	+/- 30 d	+/- 30 d	+/- 30 d
**P****h****ysical examination**	**X**	**X**	**X**	**X**	**X**	**X**	**X**	**X**	**X**	**X**
**Vital signs, weight**	**X**	**X**	**X**	**X**	**X**	**X**	**X**	**X**	**X**	**X**
**Cardiac evaluation /Echo**				**X**		**X**		**X**	**X**	**X**
**Pulmonary function/ Lufu**				**X**		**X**		**X**	**X**	**X**
**B****l****ood count/ WBC diff.**	**X**	**X**	**X**	**X**	**X**	**X**	**X**	**X**	**X**	**X**
**Questionnaires ** (EORTC QLQ-C30 & QLQ-MY20)		**X**	**X**	**X**		**X**		**X**	**X**	**X**
**B****o****n****e marrow investigation** (including MRD analysis at the UKSH central lab Kiel)		**X**		**X**		**X**		**X**	**X**	**X**
**Blood b****i****oc****h****e****m****istry** ^a^	**X**	**X**	**X**	**X**	**X**	**X**	**X**	**X**	**X**	**X**
**Serum free light chain assay and serum electrophoresis**	**X**	**X**	**X**	**X**	**X**	**X**	**X**	**X**	**X**	**X**
**Serum and urine immunofixation**	**X**	**X**	**X**	**X**	**X**	**X**	**X**	**X**	**X**	**X**
**Free light chains (24h urine)** ^**b**^	**X**	**X**	**X**	**X**	**X**	**X**	**X**	**X**	**X**	**X**
**Response evaluation** ^c^	**X**	**X**	**X**	**X**	**X**	**X**	**X**	**X**	**X**	**X**
**Adverse events (AEs)/ Serious Adverse Events (SAEs)**	**X**	**X**	**X**	**X**	**X**	**X**	**X** ^**d**^	**X** ^**d**^	**X** ^**d**^	**X** ^**d**^
**Study and Concomitant Medication**	**X**	**X**	**X**	**X**	**X**	**X**	**X**	**X**	**X**	**X**

^a^Creatinine, electrolytes, GOT, GPT, LDH, alkaline phosphatase, gamma GT, total bilirubin, uric acid, quantitative immunoglobulin, electrophoresis and total protein, GFR

^b^Exclusively in light chain myeloma (kappa or lambda type)

^c^Peripheral blood and bone marrow (bone marrow only at 100 days, 12 months, 24 months, 36 months, 48 months and 60 months)

^d^SAE reporting to the Safety Department of the CRO only takes place if the SAE is presumably related to the investigational medicinal product

## Appendix 2

**Table 6:** International Myeloma Working Group (IMWG) Uniform Response Criteria for Multiple Myeloma

Response	IMWG criteria
sCR	CR as defined below plus normal FLC ratio and absence of clonal cells in bone marrow^a^ by immunohistochemistry or immunofluorescence^b^
CR	Negative immunofixation on the serum and urine and disappearance of any soft tissue plasmacytomas and < 5% plasma cells in bone marrow^a^
VGPR	Serum and urine M-protein detectable by immunofixation but not on electrophoresis or > 90% reduction in serum M-protein plus urine M-protein level < 100 mg/24 h
PR	> 50% reduction of serum M-protein and reduction in 24 hours urinary M-protein by >90% or to < 200 mg/24 h If the serum and urine M-protein are unmeasurable,^c^ a > 50% decrease in the difference between involved and uninvolved FLC levels is required in place of the M-protein criteria If serum and urine M-protein are not measurable, and serum free light assay is also not measureable, > 50% reduction in plasma cells is required in place of M-protein, provided baseline bone marrow plasma cell percentage was > 30% In addition to the above listed criteria, if present at baseline, a > 50% reduction in the size of soft tissue plasmacytomas is also required
MR	NA
No change/Stable disease	Not meeting criteria for CR, VGPR, PR, or progressive disease
Plateau	NA
Progressive disease^c^	Increase of > 25% from lowest response value in any one or more of the following: • Serum M-component and/or (the absolute increase must be > 0.5 g/dL)^d^ • Urine M-component and/or (the absolute increase must be > 200 mg/24 h) • Only in patients without measurable serum and urine M-protein levels; the difference between involved and uninvolved FLC levels. The absolute increase must be > 10 mg/dL • Bone marrow plasma cell percentage; the absolute percentage must be > 10%^e^ • Definite development of new bone lesions or soft tissue plasmacytomas or definite increase in the size of existing bone lesions or soft tissue plasmacytomas • Development of hypercalcaemia (corrected serum calcium > 11.5 mg/dL or 2.65 mmol/L) that can be attributed solely to the plasma cell proliferative disorder
Relapse	Clinical relapse requires one or more of:Direct indicators of increasing disease and/or end organ dysfunction (CRAB features).^d^ It is not used in calculation of time to progression or progression-free survival but is listed here as something that can be reported optionally or for use in clinical practice • Development of new tisSAE plasmacytomas or bone lesions • Definite increase in the size of existing plasmacytomas or bone lesions. A definite increase is defined as a 50% (and at least 1 cm) increase as measured serially by the sum of the products of the cross-diameters of the measurable lesion • Hypercalcemia (> 11.5 mg/dL) [2.65 mmol/L] • Decrease in haemoglobin of > 2 g/dL [1.25 mmol/L] • Rise in serum creatinine by 2 mg/dL or more [177 mmol/L or more]
Relapse from CR^c^ (To be used only if the end point studied is DFS)^f^	Any one or more of the following: • Reappearance of serum or urine M-protein by immunofixation or electrophoresis • Development of > 5% plasma cells in the bone marrow^e^ • Appearance of any other sign of progression (i.e., new plasmacytoma, lytic bone lesion, or hypercalcaemia)

Adapted from Durie BGM, et al. Leukemia 2006; 20: 1467-1473; and Kyle RA, Rajkumar SV. Leukemia 2008;23:3-9

*Note:* A clarification to IMWG criteria for coding CR and VGPR in patients in whom the only measurable disease is by serum FLC levels: CR in such patients is defined as a normal FLC ratio of 0.26?1.65 in addition to CR criteria listed above. VGPR in such patients is defined as a >90% decrease in the difference between involved and uninvolved free light chain (FLC) levels

^a^Confirmation with repeat bone marrow biopsy not needed

^b^Presence/absence of clonal cells is based upon the kappa/lambda ratio. An abnormal kappa/lambda ratio by immunohistochemistry and/or immunofluorescence requires a minimum of 100 plasma cells for analysis. An abnormal ratio reflecting presence of an abnormal clone is kappa/lambda of > 4:1 or < 1:2

^c^All relapse categories require two consecutive assessments made at any time before classification as relapse or disease progression and/or the institution of any new therapy. In the IMWG criteria, CR patients must also meet the criteria for progressive disease shown here to be classified as progressive disease for the purposes of calculating time to progression and progression-free survival. The definitions of relapse, clinical relapse and relapse from CR are not to be used in calculation of time to progression or progression-free survival

^d^For progressive disease, serum M-component increases of >1 gm/dL are sufficient to define relapse if starting M-component is >5 g/dL

^e^Relapse from CR has the 5% cut-off versus 10% for other categories of relapse

^f^For purposes of calculating time to progression and progression-free survival, CR patients should also be evaluated using criteria listed above for progressive disease

## Abbreviations

AE: Adverse Event
Allo SCT: Allogeneic stem cell transplantation
Auto SCT: Autologous stem cell transplantation
AP: Alkaline phosphatase
CAR: Chimeric antigen receptors
CR: Complete response
CRAB: Calcium evaluation/ renal failure/ anemia/ bone lesions
CTCAE: Common Terminology Criteria of Adverse Events
DLCO: Diffusion capacity of carbon monoxide
DSMB: Data Safety Monitoring Board
DQB: HLA class II beta chain paralogue
Echo: Echocardiography
ECOG: Eastern Cooperative Oncology Group
EFS: Event free survival
Gamma GT: Gamma glutamytransferase
G-BA: Gemeinsamer Bundesausschuss (engl. Federal Joint Committee)
GFR: Glomerular Filtration Rate
GOT: Glutamate Oxaloacetate Transaminase
GPT: Glutamate Pyruvate Transaminase
GvHD: Graft-versus-host disease
HLA: Human leukocyte antigen
ICH-GCP: International Conference on Harmonization of good clinical practice
IMP: Interventional medicinal product
IMWG: International Myeloma Working group
ITT: Intention-to-treat
LDH: Lactate dehydrogenase
Lufu: Pulmonary function test
MM: Multiple myeloma
MMUD: Mismatched unrelated donor
MUD: Matched unrelated donor
NRM: Non-relapse mortality
OS: Overall survival
PD: Progressive disease
PR: Partial response
PFS: Progression-free survival
SAE: Serious adverse event
SAP: Statistical analysis plan
SAS: Statistical Analysis System
SD: Stable disease
SmPC: Summary of product characteristics
SUSAR: Suspected unexpected serious adverse reaction
VGPR: Very good partial response
